# Effective food waste management model for the sustainable agricultural food supply chain

**DOI:** 10.1038/s41598-024-59482-w

**Published:** 2024-05-04

**Authors:** Yuanita Handayati, Chryshella Widyanata

**Affiliations:** School of Business and Management (SBM), Bandung Institute of Technology, Bandung, 40116 Indonesia

**Keywords:** Sustainability, Environmental social sciences

## Abstract

The extensive research examines the current state of agricultural food supply chains, with focus on waste management in Bandung Regency, Indonesia. The study reveals that a significant proportion of food within the agricultural supply chain goes to waste and discusses the various challenges and complexities involved in managing food waste. The research presents a conceptual model based on the ADKAR change management paradigm to promote waste utilization, increase awareness and change people's behaviors. The model emphasizes the importance of creating awareness, fostering desire, providing knowledge, implementing changes, and reinforcing and monitoring the transformation process. It also addresses the challenges, barriers, and drivers that influence waste utilization in the agricultural supply chain, highlighting the need for economic incentives and a shift in public awareness to drive meaningful change. Ultimately, this study serves as a comprehensive exploration of food waste management in Bandung Regency, shedding light on the complexities of the issue and offering a systematic approach to transition towards more sustainable waste utilization practices.

## Introduction

The food industry comprises roughly 30% of the world’s total energy consumption, and when there is food loss and waste, the resources invested in food production go to waste^1^. Consequently, this contributes to the depletion of natural resources. Additionally, approximately 22% of greenhouse gas emissions, which have adverse environmental effects and contribute to global warming, originate from these food sectors^2,3^. To address this challenge, the United Nations has integrated the problem of food wastage into the 2030 Agenda for Sustainable Development, specifically under Sustainable Development Goal 12, which focuses on promoting responsible consumption and production. Sustainable Development Goal 12 serves as a pivotal initiative to steer away from irresponsible resource utilization and mitigate harmful effects on the planet.

Food waste management can be categorized into two main approaches: preventing the generation of waste and handling waste that has already been produced^4^. The strategies for implementing food waste management vary depending on the underlying causes of each specific food waste scenario. This is because food loss and waste can manifest differently and require distinct treatments or solutions, occurring at various stages of the supply chain, ranging from production upstream to consumption downstream^5^. Efforts to address these issues can also be observed within different stages of the supply chain. For instance, at the production stage, optimization of production factors, such as infrastructure improvements^6^ or the use of forecasting to prevent overproduction^7^ is emphasized. In the distribution stage, enhancing efficiency in the distribution process can be achieved by shortening the supply chain^8^ or by fostering coordination among supply chain participants^9,10^. Similarly, at the consumption stage, efforts often focus on enhancing supply chain processes to increase efficiency by utilizing waste to create more valuable product, ultimately reducing waste, which is commonly referred to as waste prevention.

Many nations worldwide have embraced the United Nations' objectives of minimizing food waste and promoting sustainability, demonstrating a collective dedication to addressing crucial environmental and social issues. The UN's Sustainable Development Goal 12 emphasizes the importance of curbing food waste across supply chains. This has spurred countries to take tangible steps and enforce policies aimed at reducing food waste^11^. For instance, the European Union (EU) has committed to ambitious targets outlined in its Circular Economy Action Plan to slash food waste by 2030. Strategies such as standardized date labeling, awareness campaigns, and support for surplus food donation align with the UN's sustainability agenda. Similarly, nations like South Korea have implemented innovative approaches, including pricing based on waste volume, mandatory food waste separation, and promoting the conversion of food waste into compost or biogas. These initiatives not only resonate with sustainability goals but also contribute to mitigating greenhouse gas emissions.

Furthermore, scholarly research available in publications such as "Resources, Conservation & Recycling" and "Waste Management" investigates the impact of diverse national policies on food waste reduction and sustainability. These studies analyze the effectiveness of specific interventions and offer insights into successful strategies adopted by different countries. By citing these examples and research outcomes, one can illustrate how nations are actively aligning themselves with the UN's aims of reducing food waste and promoting sustainability through a combination of policy frameworks and practical implementations.

In relation to that, food waste pre-treatment technologies have also been extensively developed to reduce the carbon loss as Carbon dioxide during storage/transport; improve the surface properties for easier access to microbes; (reduce the accumulation of volatile fatty acids at early stages or during storage and transport; and alter biological properties to support microbiomes from anaerobic digestion / dark fermentation^15,16^. This pre-treatment can be carried out either through physical and mechanical pre-treatments, Thermal pre-treatment, Chemical pre-treatment and Biological pre-treatment^16^. Nevertheless, landfilling of food waste is a very common disposal method in developing countries e.g., India, China, Thailand, Bangladesh, Sri Lanka, etc. It is due to their national budget for waste management. Due to insufficient funding for recycling, some developing nations have attempted to introduce a system for managing food waste in their legislative frameworks. However, budgeting remains a significant problem in developing countries for handling waste^17,18^^.^

Indonesia faces significant food waste issue, with food waste accounting for 28.6% of total waste. To address this problem, the government has outlined plans in its 2020–2024 National Mid-Term Development Plan to reduce waste by up to 80%^19^, including food waste. The Ministry of Agriculture’s Strategic Plan for 2020–2024 and The Indonesia Food Sustainable System 2019 further emphasize efforts to combat food waste by following decentralized approach, giving local goverments the authority to manage related issue. This approach encourages collaboration among all stakeholders, both nationally and locally^20^. Notably, the Bandung Regency government is one local authority actively addressing food waste. ^20^2019 To address this issue, Development Agency at Sub National Level is actively working on establishing a more sustainable food supply chain for the implementation in Bandung Regency. In the context of advancing food security in Bandung Regency, the government’s strategy consists of five core concepts, encompassing food supply chain efficiency, connectivity, price regulation, logistics cost reduction, enhanced production capacity, and sustainability. This sustainability aspect also encompasses initiatives related to waste processing, as outlined by Bappeda Kabupaten Bandung^21^. The latest attempt in developing a sustainable supply chain in Bandung Regency is the establishment of a food hub is an endeavor by the government to build a more efficient supply chain, which is described as an aggregator capable of integrating all parties involved, acting as a logistical service provider, marketing, agricultural product added-value development, and information hub^22^.

^23^When considering the five key concepts for enhancing food security in Bandung Regency, the establishment of the food hub addresses four of these concepts, primarily focusing on waste prevention. However, there is a notable absence of detailed research or government reports that specifically address the fifth concept, which pertains to sustainability and effective management of existing food waste. As previously mentioned, one of the primary contributors to the increasing waste issue is the lack of proper handling of generated waste. Furthermore, the linear economy approach, which categorizes all unused products as waste, exacerbates the problem. Additionally, the growing population is a factor leading to increased waste, while the landfill capacity remains limited. Hence, while waste prevention is crucial, there's still a pressing need for well-planned food waste management, particularly in terms of waste utilization because waste can be utilized wisely to make it more valuable^23^. To optimize waste utilization, it is imperative to develop a comprehensive waste management strategy to avoid the oversight of waste reduction^24^. This strategic planning encompasses the crucial step of waste identification, involving the collection of data regarding the types of waste, the locations where waste is generated, and potential methods for waste utilization^19,24^. Understanding the composition and sources of waste will greatly facilitate effective waste management^25^.

Currently, there is no available data or research on food waste management in the Bandung Regency's Food Supply Chain. This study aims to address this gap by identifying food waste in the region's supply chain, with the goal of promoting the development of a more sustainable food supply chain. Therefore, this study aims to develop effective food waste management that can be implemented in Bandung Regency’s food supply chain. In addition, study by Nattassha et al.^26^ emphasized the importance of integrating waste management actors, including scavengers, sorters, and processors, with resource suppliers and producers to facilitate the reuse of treated waste. This study collected data from these stakeholders to enhance understanding and proposed a conceptual model to improve waste management knowledge among producers. It advocates for a comprehensive approach involving all actors in food waste management, which hasn't been previously explored.

## Results

### The real-world situation of food supply chain in Bandung Regency

The supply chain at Bandung Regency involves three primary participants: farmers, intermediaries, and customers. Each of these actors assumes distinct roles and responsibilities within the agricultural product supply chain. Farmers are individuals responsible for producing agricultural products. Intermediaries are entities that aid farmers in the distribution of their products to the primary consumers. These intermediary participants can be categorized into two groups: wholesalers and retailers. Wholesalers are entities that acquire these products from farmers, either directly or indirectly, and subsequently sell them to purchasers in bulk quantities. Meanwhile, retailers are parties who directly sell products to the end consumers^27^.

There are two categories of wholesalers: merchant wholesalers and agents or brokers. The distinction between a merchant wholesaler and an agent or broker is found in how they participate in the supply chain process of distributing goods. Agents or brokers primarily facilitate connections between farmers and wholesalers who have direct market or customer access. They do this through communication and negotiation without physically handling the agricultural products, a role often referred to as being intermediaries or middle-men^27,28^. On the other hand, supermarkets are larger, modern retailers with a self-service concept, aiming to fulfill consumers' complete grocery and household product needs^27^. Online retailers conduct transactions without the need for physical interaction between sellers and buyers, operating through online platforms.

Lastly, customers are individuals or entities that use or consume the agricultural products, either for personal use or for further distribution as different products. In the agricultural product supply chain within Bandung Regency, customers can be categorized into two groups based on how they utilize the purchased items: the consumer market and the business market^27^. Consumer markets involve individuals who use products for personal consumption, while business markets consist of customers who purchase and distribute products in bulk, often to other businesses or consumers after processing.

**Figure 1:**
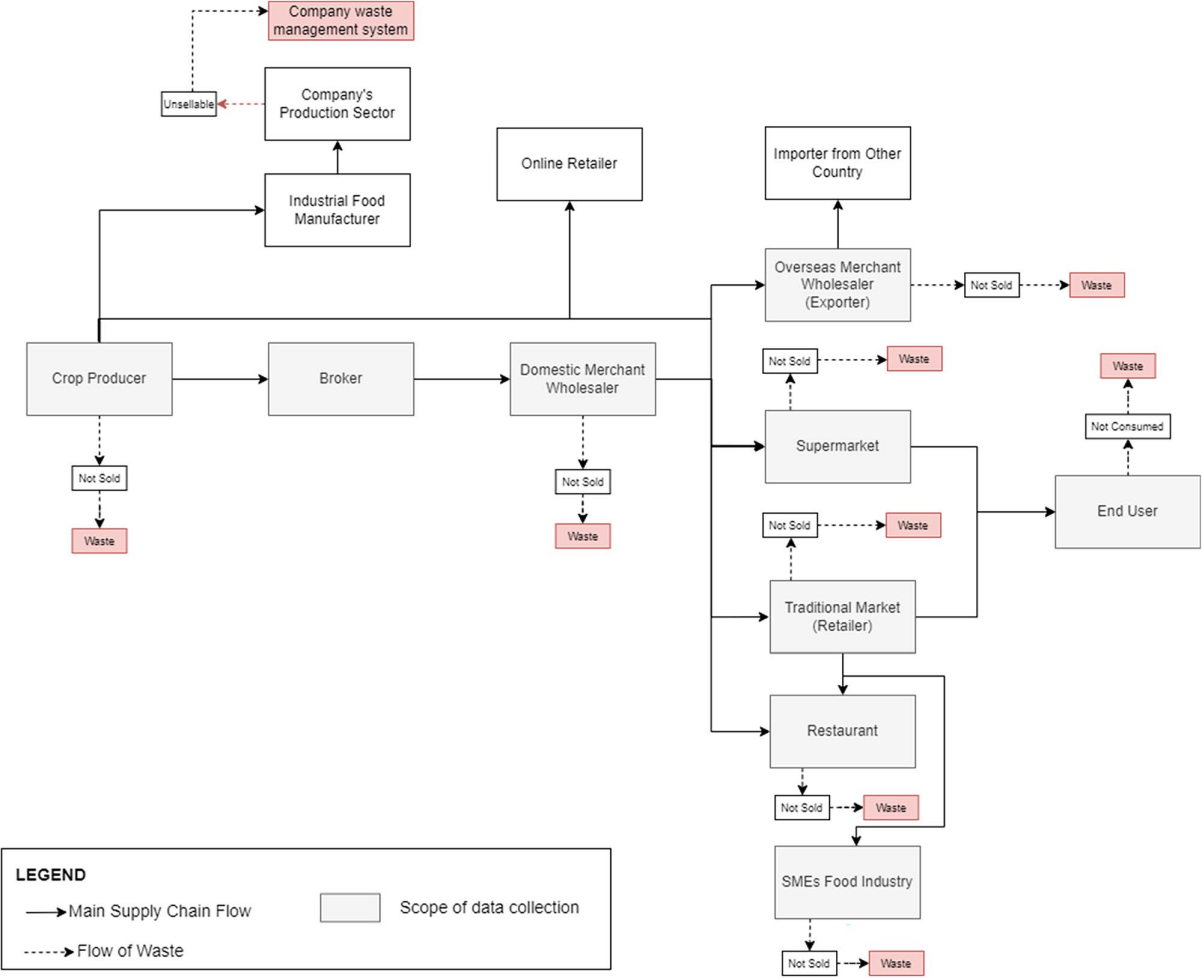
Bandung Regency’s Current Agricultural Supply Chain.

Figure 1 illustrates the movement of agricultural products, particularly vegetables and fruits, within the agricultural supply chain of Bandung Regency. The figure depicts that agricultural products have their source in farmers or crop producers and ultimately reach consumers, encompassing both business clients and individual end users.

### Current handling of unused product during the supply chain process

While it may seem that agricultural products follow a path from farmers as producers to eventual consumers, not all of these products find buyers and are sold. According to the data gathered, a significant portion of unsold products ends up as waste. Interestingly, not all of these products are in poor condition, and some still possess quality suitable for sale in the market. These unsold products can be categorized into three broad groups based on their condition, as outlined in the matrix proposed by Teigiserova, Hamelin, and Thomsen^29^: surplus food, food waste, and food loss.

**Figure 2:**
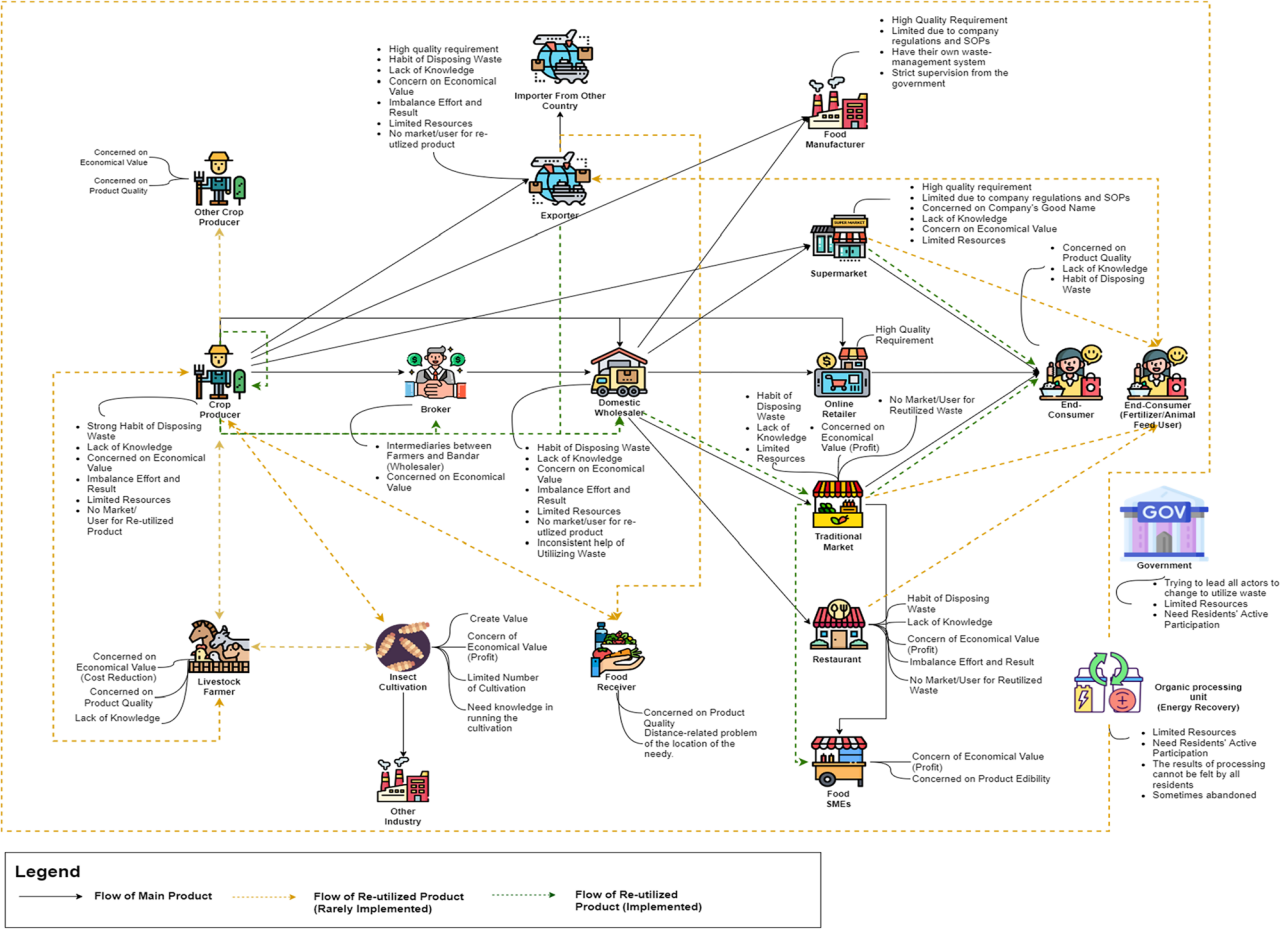
Rich Picture of Bandung Regency’s Agriculture Supply Chain and Current Waste Management Practice.

To reduce food surplus, the "reduce" principle can be applied through measures like careful production planning or the utilization of advanced storage technologies, such as cold chain management. As per the interviews, certain actors, particularly those in financially stable positions like supermarkets, exporters, and restaurants, have successfully implemented waste reduction efforts, and the outcomes have indeed assisted them in waste reduction. However, some other actors still face challenges in implementing these measures, primarily due to limited financial resources (additional obstacles can be found in Fig. 2, the Rich picture).

The "reuse" principle, particularly for surplus edible products, is crucial alongside prevention measures. Common methods include distributing to food collection organizations, providing to local communities for free, selling at reduced prices, and processing into other food items. Selling at lower prices is the most commonly adopted. Partially edible products are often reused, while true food waste can be repurposed through recycling for animal feed, composting, insect rearing, and material recovery. However, recycling efforts are limited due to a lack of knowledge, leading some to dispose of unused products. Another option is energy generation through anaerobic digestion, but it's currently underutilized.

Meanwhile, according to government officials interviewed, it was emphasized that independent waste management efforts by the community were essential. This was seen as necessary because it would be impossible for the government alone to handle all waste-related responsibilities. A key limitation from the government's perspective is the inadequate waste management infrastructure in Bandung Regency.

As stated in the 2018 performance report of the Bandung Regency Environmental Service, with only 100 waste transport vehicles, the government was able to collect and transport a mere 16.32% of the waste, a figure that decreased further in 2019 to 12.6% due to a rise in waste generation. Consequently, the Bandung Regency government encourages residents to take a more active role in waste management.

The government has initiated various efforts to enable citizens to participate in waste reduction. However, in practice, people have been slow to embrace waste management practices. Even with organizational support, only 40% of the population actively engages in these programs, as per representatives from non-governmental organization s during telephone interviews on June 16, 2022. Additionally, when not continuously supported, people tend to discontinue their participation. Meanwhile, the organizations themselves face resource limitations, preventing them from providing ongoing assistance and monitoring to residents. The challenges faced by various actors and their competing priorities often lead them to opt for waste disposal rather than utilization. Figure 2, the Rich Picture, illustrates the complex issues within the agricultural product supply chain in Bandung Regency and waste management.

### Root definition

The Rich Picture diagram illustrates that actors have not fully embraced waste utilization. Despite the obstacles and concerns expressed in interviews, the main challenge lies in changing people's ingrained habit of disposing of anything they consider useless. Society is accustomed to discarding items, while the government aims to encourage people not to waste potentially useful items and find ways to repurpose them. This is a significant hurdle as these habits have persisted for a long time and are deeply ingrained. When asked why they don't utilize waste, some individuals couldn't provide specific reasons and considered discarding waste as an automatic and unquestioned habit.

However, other barriers contribute to people's reluctance to utilize waste. Interviews reveal that a common obstacle is the lack of public awareness about the significance and urgency of waste issues, as well as limited knowledge about waste management. Many interviewees indicated that they hadn't experienced any negative consequences from waste accumulation, and some considered littering as a normal practice driven by their circumstances.

The issue of low public awareness of waste problems is also acknowledged by government agencies and non-governmental organization’s working in the solid waste sector. The abandonment and limited success of various waste reduction programs and facilities can be attributed to this problem. As mentioned earlier, even when the government and non-governmental organization’s assisted communities in implementing waste reduction programs, these initiatives were not adopted by 100% of the residents, and often not even by half of them. This drop-off in participation occurred particularly when residents were no longer under active supervision, despite initially appearing proficient in executing the programs during mentoring periods. Consequently, the model areas or waste processing assistance efforts were not sustained, and residents reverted to their old habits. (Non-governmental organization Representatives, Telephone Interview, 16/06/2022).

Waste can be used wisely to make it more valuable. Certain agricultural products such as fruit remnants can be repurposed into other valuable products by recovering their bioactive compounds through valorization techniques^23^. Some individuals have attempted to reuse waste by processing it into fertilizer, selling it in the market, or transforming it into other products. However, the outcomes often did not justify the effort expended, leading them to revert to discarding waste. The comparison between results and effort involved revolves around the processed products' energy, time, and additional costs required for waste processing. For example, energy generated from waste processing in a biodigester was only sufficient for 1-2 nearby houses or a community meeting hall, indicating limited impact.

The economic value of waste utilization presents as second obstacle. While some individuals are willing to utilize waste for economic benefits, many view its main advantage as environmental. This perspective is especially common among economically disadvantaged individuals. Market challenges, such as distance from potential users and a lack of awareness about product benefits, also hinder waste utilization. Additionally, farmers may continue to harvest even in oversupplied markets, leading to increased costs and waste. This economic focus discourages waste processing.

The third obstacle is limited resources, such as time, funding, manpower, and technology. Time constraints are the major issue, as supply chain actors prioritize their core income-generating activities. Financials limitations, especially among unstable actors, hinder investments in technologies like cold storage or food processing tools.

Supermarkets, in particular, face space limitations for waste processing, and these constraints can lead to discontinuation of waste utilization programs in favor of waste disposal through cleaning services. Overall, changing waste management habits is challenging when immediate waste disposal is the norm, and public awareness of the government's goals is lacking. Perceived benefits, distribution challenges, and resource limitations further deter habit changes. A CATWOE analysis, aimed at shifting waste handling habits towards waste utilization, is detailed in the table below.

The Table 1 CATWOE analysis shows how the ideal system is to produce an effective transition to the habit of utilizing waste.In the CATWOE framework, the first element is the "customer," which, in this context, refers to society at large within the agricultural supply chain. The second element, the "actor," encompasses all stakeholders committed to changing food waste disposal habits. Collaboration is essential to effectively bring about this change. The third element, "transformation," aims to change habits while considering the factors driving and inhibiting change. The fourth element, "Weltanschauung," emphasizes that this change system should align with individuals' fundamental needs for achieving and sustaining change. The "owner," as the fifth element, is the government, which not only acknowledges the food waste issue but also holds the authority to influence and regulate societal behavior. The final element, the "environment," encompasses the entire agricultural product supply chain, extending beyond Bandung Regency.

**Table 1:** CATWOE Analysis.

Effective Transition to The Habit of Utilizing Waste	
Customer	Society (which includes all food supply chain actors)
Actor	Government, educational and research institution, non-governmental organizations , Society
Transformation	Transitioning new habit by considering the driver and barriers of change
World-wide view	Changing habit can be effectively carried out by considering the basic requirement of a person to achieve and sustain change
Owner	Government
Environment	Agriculture Supply Chain

### Conceptual model

The CATWOE analysis indicates a need for a mechanism to enhance how people utilize waste. To address this, a conceptual model was developed in this study, utilizing the ADKAR change management paradigm, which was introduced by Prosci in 1998. The selection of the ADKAR model was based on its appropriateness for implementing changes that require acceptance from those undergoing the change, in this case, society. This choice was made considering the scope and impact of the change. Therefore, Fig. 2, titled "The Conceptual Model," illustrates the system for altering people's behaviors to maximize waste utilization.

**Figure 3:**
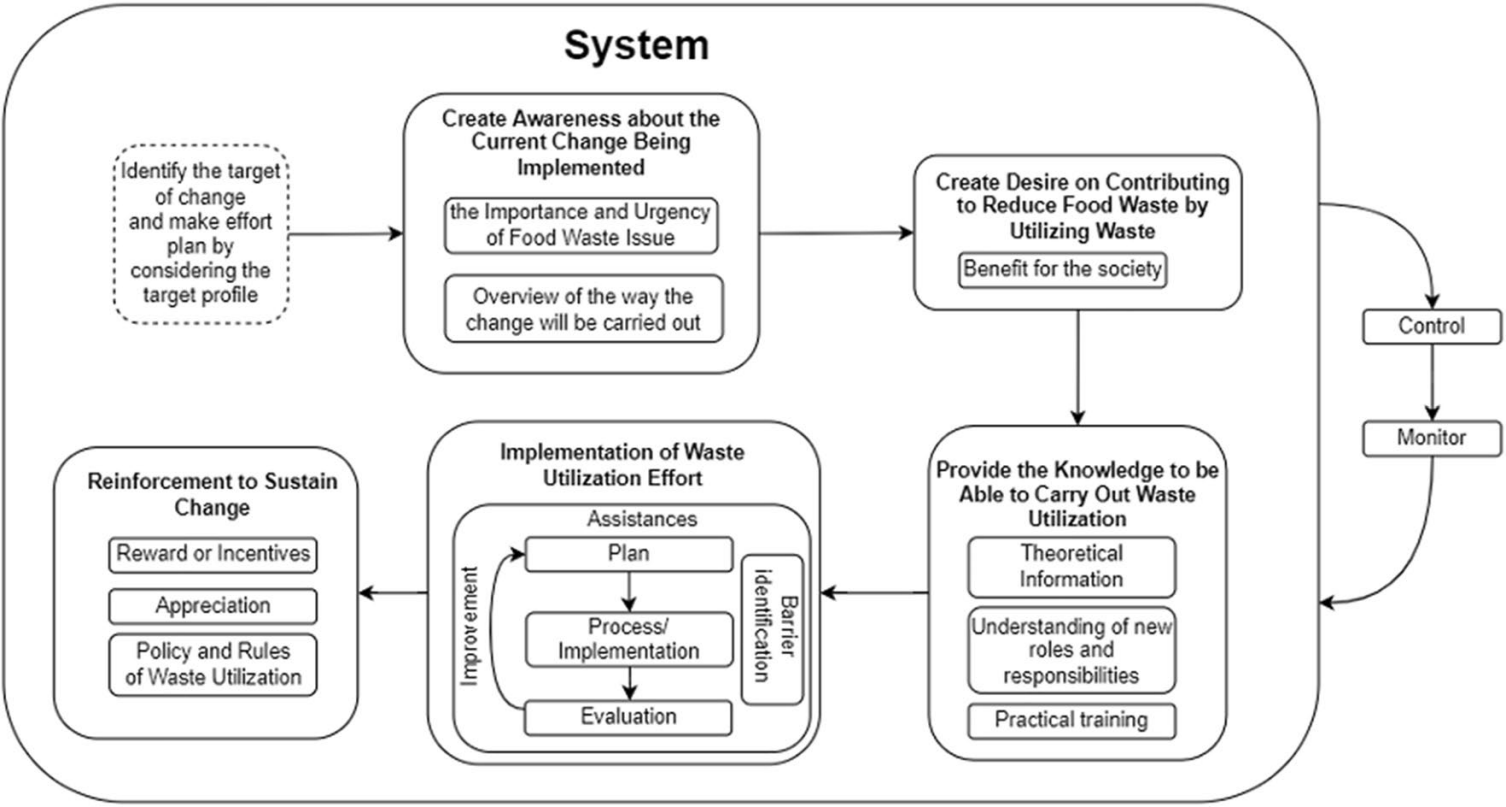
The Conceptual Model.

According to the ADKAR model in Figure 3, the first step in facilitating change is to create awareness among those involved. This awareness should encompass an understanding of the reasons for change and the potential risks if change is not implemented. In the context of promoting waste utilization^30^, it's crucial for change agents to ensure that people comprehend the issues surrounding food waste and how utilizing waste can address these concerns. Without this understanding, people may be hesitant to change their habits. The subsequent step in driving change is to stimulate people's desire to use waste, as this motivation is what can encourage active participation in the change process. In the context of waste utilization, change agents must grasp the community's desires and needs regarding waste use to motivate them for necessary changes. However, the lack of perceived benefits from changing routines has hindered supply chain actors' embrace of waste utilization. Interviews with those who have used waste revealed a positive impact, especially on environmental aspects, but this alone wasn't enough motivation to continue, except for individuals in supermarkets who viewed environmental concerns as part of their corporate social responsibility. Their primary focus, though, was on economic aspects. In fact, most respondents indicated that they would be more interested in waste utilization if processed waste products could provide economic value by increasing income or reducing expenses.

The next step involves changing people's behavior by providing them with information on effective waste utilization. This goes beyond theoretical knowledge and includes practical understanding of the new tasks and responsibilities associated with these changes, along with training. Four key aspects must be addressed when influencing change knowledge: existing community knowledge, the community's learning capacity, available resources for education and training, and access to information. It's crucial to consider these factors for effective knowledge delivery. Change agents should tailor their approach to the specific audience they are addressing.

Once the community has the necessary knowledge, the next phase is to implement waste utilization. This phase includes developing strategies and action plans and evaluating the effectiveness of implementation. Putting knowledge into practice is vital because theory and practice can differ. To sustain these changes, reinforcement is essential. This can be achieved through incentives, recognition, or even government policies mandating the changes. Finally, change agents must continuously monitor and control their efforts to alter waste utilization habits, understanding that forming new habits takes time, especially in large-scale changes. Monitoring and control ensure alignment with government objectives and allow for necessary adjustments.

The ADKAR model outlined in the context of waste utilization provides a structured approach to driving change by focusing on awareness, desire, knowledge, action, and reinforcement. The applicability and effectiveness of the model in the context of waste utilization depend on its successful adaptation to local contexts, effective stakeholder engagement, practical knowledge delivery, and ongoing monitoring and reinforcement efforts. When implemented thoughtfully and comprehensively, the model can serve as a valuable framework for driving sustainable change in waste management practices.

## Discussion

Based on the issues outlined in the root definition and conceptual model, it's evident that those driving change must initially focus on raising awareness and fostering a desire for the intended change. However, it's crucial to emphasize that planning these efforts should not be divorced from setting specific change objectives in advance to ensure that these endeavors stay on course. The first approach to achieve this is through expansion.

Factors such as economic conditions, income levels, and the cost associated with waste disposal services significantly affect individuals' decisions about managing their waste^31^. In addition, sociocultural beliefs, societal norms, and perceptions regarding waste disposal practices also play a crucial role in waste management^32^. Moreover, individual behaviors, preferences, and levels of environmental consciousness significantly influence how people dispose of their garbage^33^.

Therefore, expansion is needed to raise awareness and shift people's perspectives about waste. The intention is to strengthen their knowledge in waste management and its impact. According to research by McCoy^34^, the role of expansion is to alter how people perceive and manage something, in this case, food waste. Collaborating with broad array of experts and stakeholders offers an opportunity to enhance education and understanding of food waste, serving as a foundation for instigating habitual changes towards its utilization.

Behavioral science research, exemplified by Cialdini's on social influence and persuasion underscores the significance of comprehending human behavior to shape attitudes and encourage the adoption of new practices. Employing principles from behavioral psychology can assist in devising interventions that advocate for the adoption of effective waste disposal methods^35^. Therefore, involving the community in decision-making processes concerning waste management interventions instills a sense of ownership. Studies like those conducted by Lockwood et al. highlight the importance of community engagement and participatory approaches in waste management initiatives, resulting in enhanced acceptance and sustainability of implemented measures^36^

Efficient communication and educational campaigns are instrumental in gaining public support and comprehension. Research by Maibach et al. emphasizes the significance of targeted communication strategies in facilitating behavioral changes related to environmental issues, including waste management^37^.

Therefore, utilizing social media as a educational campaign tool to raise public awareness is one viable method to create more efficient communication. Given the continuous growth in the number of internet and social media users in Indonesia, social media can be an effective medium for disseminating information to enhance public awareness. According to research by Jenkins et al.^38^, social media has demonstrated a positive impact on raising awareness and contributing to the reduction of food waste, particularly at the consumer level. In addition to the awareness issue, it was previously noted that another challenge is the perceived lack of benefits by society. Nattassha et al.'s^26^ research highlights the critical role of incentives in encouraging cassava supply chain growers to adopt a circular economy, thereby motivating them to remain engaged in the supply chain. Presently, the benefits expected by the community are linked to the economic value of waste disposal. The establishment of a circular economy represents one strategy to align people's desires with waste utilization.

Further, intelligence and digitalization play a crucial role in shaping an effective waste management model. These approaches can offer several advantages, such as real-time monitoring of waste collection, optimizing routes for garbage trucks, and improving recycling processes through data analysis^39^. Studies published in journals like "Separation and Purification Technology" often explore the realm of intelligent waste management systems. These systems utilize digital technologies such as IoT (Internet of Things), AI (Artificial Intelligence), and data analytics to streamline waste collection, recycling procedures, and resource allocation. For instance, Babaei and Basu^40^ delve into the implementation of IoT and AI in waste management in their work^40^.

Additionally, research by Tao et al.^41^ utilize 20-kHz ultrasound, this study extracted phenolics from Chinese chokeberry using distilled water and 50% aqueous ethanol, revealing that adaptive neuro-fuzzy inference system (ANFIS) successfully correlated extraction parameters with high total phenolic yield, while identifying the effectiveness of different solvents for extracting specific phenolic compound.

Technological progress holds a significant role in waste management. Progress in waste-to-energy technologies, recycling processes, and intelligent waste management systems profoundly affects the effectiveness and sustainability of waste management practices^42^. Therefore, continuous evolution of policies is essential, taking into account technological advancements, socio-economic changes, and environmental considerations. This flexibility and adaptability within policies are crucial to ensure the effectiveness and relevance of waste management strategies amidst changing circumstances and emerging challenges.

## Conclusion

In conclusion, effective communication and educational campaigns, including the use of social media, can enhance public awareness and understanding of waste management. Furthermore, implementing a circular economy and integrating intelligence and digitalization into waste management systems are crucial for improving their effectiveness and sustainability. However, the study has limitations. It focuses solely on Bandung Regency, potentially limiting the generalizability of its findings. Additionally, constraints related to data availability and resources, as well as the complexity of interdisciplinary approaches to waste management, may impact the research. Future studies should address these limitations by conducting comparative studies across different regions to identify variations in waste management practices. Longitudinal studies are also needed to assess the long-term effectiveness of interventions and monitor changes in waste management behaviors over time. Additionally, exploring innovative approaches to enhance community engagement and participation in waste management initiatives is essential.

## Methods

The research method employed in this study is Soft Systems Methodology (SSM), which was developed by Checkland in 1989. The choice of this methodology is based on its suitability for addressing the research questions, considering the study's context and subject matter. This research aims to identify the current management of food waste and the potential for food waste utilization in Bandung Regency, with a focus on waste flow within the agricultural supply chain. The study involves gathering insights from various stakeholders.

**Figure 4:**
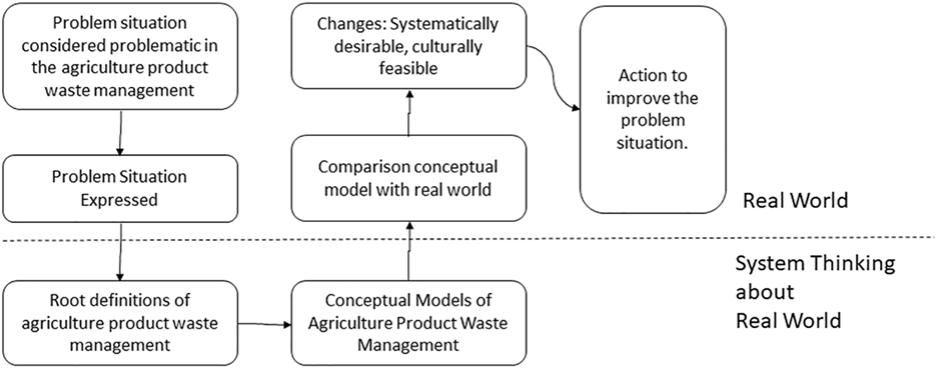
Seven Stages of SSM.

Given that this research utilizes the Soft Systems Methodology (SSM) approach, the research process follows the steps outlined by Checkland. Checkland's SSM involves a seven-stage model, and Figure 4 illustrates how the research is conducted.

Primary data is acquired through a primary semi-structured interview conducted via purposive sampling. Interviews were carried out with a total of 27 respondents who had connections to and involvement in the agricultural product waste supply chain in Bandung Regency. These respondents represented various roles, including farmers, domestic and overseas merchant wholesalers (exporters), traditional market wholesalers, retail sellers in traditional markets, supermarket representatives, restaurant managers, small and medium-sized food business owners, cattle fattening workers, chicken farmers, private agricultural extension agents, farmer cooperation representatives, public relations personnel from non-profit organizations focused on waste, government representatives, and end-users. Secondary data is sourced from existing literature.

## Data Availability

The datasets used and/or analyzed during the current study available from the corresponding author on reasonable request.
